# The relationship between HbA1c and the activities of daily living in complex chronic patients with and without intracerebral hemorrhage

**DOI:** 10.1186/s12883-024-03764-3

**Published:** 2024-09-13

**Authors:** Ying Zheng, Chenju Zhan, Qixi Liu, Chengsheng Chen

**Affiliations:** Mindong Hospital Afiliated to Fujian Medical University, Fuan, Fujian 355000 People’s Republic of China

**Keywords:** HbA1c, Complex chronic patients, Intracerebral hemorrhage, Activities of daily living

## Abstract

**Background:**

Associations between HbA1c and adverse outcomes in ischemic and hemorrhagic stroke have been confirmed. It is still unclear whether HbA1c is related to the activities of daily living (ADL) score in complex chronic patients (CCP) with and without intracerebral hemorrhage (ICH).

**Aim:**

The associations between HbA1c and ADL (Barthel score) in CCP with ICH and without ICH were evaluated, respectively.

**Methods:**

We have analyzed data from a previous cohort study involving in 3594 CCPs without a ICH history at baseline, who were followed up for 5 years to assess ICH episode.

**Results:**

One hundred sixty-one ICH case were detected in a total of 3594 patients during the period of follow up for 5 years. Our nonlinear analysis suggested positive trends on the association between HBA1c and Barthel score in ICH and non-ICH patients, respectively. The multivariate linear regression analysis showed that elevated HbA1c was positively associated with a higher Barthel score among all study population (β = 1.25, 95% CI: 0.92, 1.59; *P* < 0.0001) with adjusted age and sex. Among non-ICH patients, increased HbA1c was still positively associated with an increased Barthel score (β = 1.24, 95% CI: 0.90, 1.58; *P* < 0.001). However, HbA1c appeared to have no any relationship with Barthel score in ICH patients (β = 1.87, 95% CI: -0.07, 3.82; *P* = 0.0613) after adjustment for age and sex. By additionally using sensitivity analysis, we still observed that the strong relationship was still existed in non-ICH patients (β = 0.90, 95% CI: 0.56, 1.24; *P* < 0.001) but not in ICH patients (β = 1.88, 95% CI: -0.10, 3.86; *P* = 0.0649).

**Conclusion:**

We observed for the first time that elevated HbA1c is associated with better ADL in CCPs without ICH but not in those with ICH. This interesting discovery contradicts the traditional adverse effects of elevated HbA1c.

## Introduction

Intracerebral hemorrhage (ICH) is a very serious disease condition that have been accounted for 10–15% of stroke events related to a poor prognosis [1, 2]. The global burden of disease (GBD) showed that the annual number of strokes and deaths due to stroke increased substantially from 1990 to 2019 [3], and the data on the burden of stroke from China suggested that in a nationally representative sample of adults aged 40 years or older, the estimated prevalence, incidence and mortality rate of stroke in China were 2.6%, 505.2 per 100 000 person-years and 343.4 per 100 000 person-years in 2020 respectively, indicating that an improved prevention strategy for stroke is necessary in the general Chinese population [4]. Previous studies have confirmed that diabetes mellitus is a risk factor for both ischemic and hemorrhagic stroke [5–10]. For example, a cohort study including 297,486 with diabetes and 1,167,585 without diabetes performed in Israel reported that diabetes was independently associated with increased ICH risk, with hazard ratio (HR) of 1.36 (95% CI 1.27–1.45) [5]. Among patients with diabetes, HbA1c had a nonlinear J-shaped relationship with ICH risk (P for nonlinearity = 0.0186) [5]. A recent study by a Chinese scholar also reported that admission hyperglycemia is a common question after ICH, and stress-induced hyperglycemia is a sensitive predictor of the risk for pulmonary infection and all-cause death after ICH [6]. Another retrospective study indicated that glycated hemoglobin (HbA1c) is an important predictor of symptomatic ICH after thrombolysis for acute stroke in 1112 consecutive patients treated with thrombolysis [7]. However, evidence for the association between HbA1c, a much more accurate measure of glycemic control than glucose levels, and activities of daily living (ADL) in complex chronic patients (CCP) with and without ICH is very limited now [6–10].

Here, we obtained data from a previous cohort study involving in 3594 CCPs who were followed up for 5 years to assess ICH event. Our study aimed to evaluate the association between HbA1c and activity of daily living (ADL) score calculated by Barthel score in CCP with ICH, as well as in those without ICH.

## Materials and methods

### 1Study design

We obtained the dataset (*N* = 3594) that has been deposited in the public repository (10.5061/dryad.t76hdr7zj) [11]. Briefly, this dataset was originated from a retrospective and multi-center cohort study (Terres de l’Ebre, Catalonian South) for an ICH event that were collected from April 1, 2006 to December 30, 2016, with the main aim of designing a predictive index of ICH related mortality in the community-based population. For research purposes, we included all 3594 complex chronic patients for characteristics analyses after excluding patients with missing data. We retrospectively used a cross-sectional analysis to evaluate ADL score (Barthel score) data and further analyzed its relationship with HbA1c in CCP patients with ICH (*N* = 161) and without ICH (*N* = 3433), respectively. The diagnosis for ICH was obtained from a computerized database and all other diagnoses were based on information from assessments conducted during home visits and records from medical institutions. A physician comprehensively reviewed the digital medical charts of individuals. The definition of CCP needed to meet at least four criteria according to the definition of previous study [11, 12].

All methods were performed in accordance with the relevant guidelines and regulations. The study protocol obtained ethics approval from the Ethical Committee of Jordi Gol University Institute of Primary Care Research and the informed consent for all patients were waived prior to the inclusion of their medical data in this study.

### Barthel score

The full name of the Barthel score, also known as the Barthel Index of ADL, is a widely used daily living activity assessment method in the field of rehabilitation therapy [13]. It was designed and developed by American Dorothy Barthel and Florence Mahney. The Barthel score is mainly used to evaluate the self-care ability of elderly people and patients with neurological, muscular, and skeletal diseases. It includes 10 aspects of abilities, including eating, bathing, dressing, personal hygiene, bowel and bowel control, bed and chair transfer, walking, and going up and down stairs. The rating for each project ranges from 0 points (completely dependent) to 15 points (completely independent), with a maximum total score of 100 points. According to Barthel's rating, an individual's daily living ability is divided into different levels: a score greater than 60 indicates that the individual can basically take care of themselves. A score of 40–60 is considered mild disability and requires assistance from others to complete daily activities. A score of 20–40 is considered severe disability and requires a significant degree of dependence on others for daily life. A score below 20 is considered completely disabled and requires complete dependence on others for daily life. In addition, the Barthel score can not only be used to evaluate functional status before and after treatment, but also to predict treatment efficacy, hospital stay and prognosis.

### Covariates

To achieve the objectives, relevant covariates including age, sex, CHADsVASC [congestive heart failure, hypertension, age ≥ 75y (doubled), diabetes mellitus, stroke (doubled)-vascular disease, age 65–74 and sex category (female)] score, HAS-BLED (hypertension, abnormal renal/liver function, stroke, bleeding history or predisposition, labile INR, elderly, drugs/alcohol concomitantly) scores, chronic disease [dementia cognitive impairment, diabetes, atrial fibrillation, hipercholesterolemia, ischemic cardiomyopathy, peripheral vascular disease, heart failure, thromboembolism, stroke or transient ischemic attack (TIA), chronic renal disease, chronic liver disease and neoplasia] and medication use (oral anticoagulant treatment, nonsteroidal anti-inflammatory drugs, antiaggregants and statines). Except for sex (female and male), other categorical variables including complex chronic disease and medication use were defined as “yes” or “no”, respectively.

### Statistical analysis

All statistical analyses in this study were performed by Empower version 4.1 and statistically significance was defined as P ≤ 0.05. Demographic data and complex chronic disease information in two subgroups (patients with ICH and without ICH) were firstly described. The continuous variables were presented as medians (interquartile range, IQR) or means (standard deviation, SD), and categorical variables were presented as frequencies (percentage). Then, nonlinear evaluation was performed on association between HBA1c and Barthel score by an adjusted smooth curve. Multivariate linear regression models were also fitted to evaluate the association between HBA1c and Barthel score adjusted for confounding factors: Model 1 adjusted for age, and Model 2 adjusted for age and sex described by 95% confidence intervals (CI) and correlation coefficient (β). We further performed a sensitivity analysis by adding CHADsVASC score and HASBLED score into the above models respectively. In the end, stratified analysis of CCP was further conducted to investigate the association between HBA1c and Barthel score.

## Results

### Characteristics in complex chronic patients

**Fig. 1:**
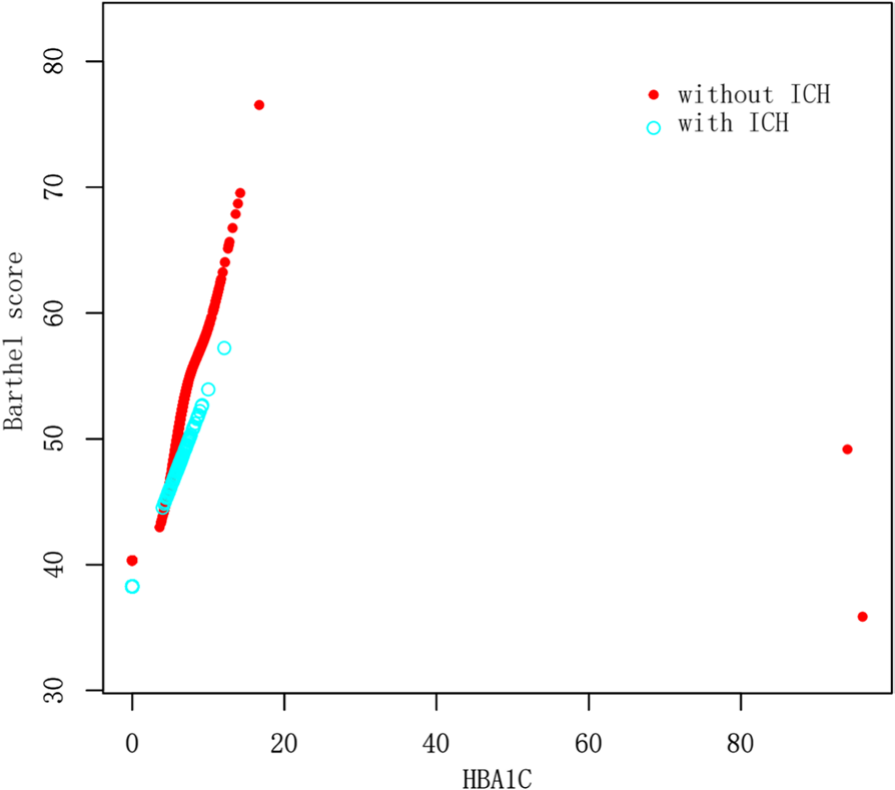
Adjusted non-linearity analysis for relationship between HbA1c level and Barthel score

One hundred sixty-one ICH events were detected in a total of 3594 patients. Then all subjects were divided into two subgroups (161 ICH patients with mean age 86.5 ± 8.7 years and 3433 non-ICH patients with mean age 84.3 ± 11.6 years) were enrolled into our study, and the both of them 90 (55.9%) and 1895 (55.2%) were male respectively. The distributions for demographic and clinical characteristics in these included individuals with ICH and without ICH were showed in Table 1. ICH patients were older with a higher rate for neoplasia and used medication (antiaggregants). There were no significant differences in sex, CHADsVASC score, HAS-BLED score, complex chronic disease (dementia cognitive impairment, diabetes, atrial fibrillation, hipercholesterolemia, ischemic cardiomyopathy, peripheral vascular disease, heart failure, thromboembolism, stroke or TIA, chronic renal disease and chronic liver disease) and medication use (oral anticoagulant treatment, nonsteroidal anti-inflammatory drugs and statines). Importantly, nonlinear analysis by an adjusted smooth curve indicated almost consistent trends on the association between HBA1c and Barthel score in two subgroup (Fig. 1).

**Table 1:** Characteristics in complex chronic patients (*N* = 3594)

Baseline variables	With ICH (*N* = 161)	Without ICH (*N* = 3433)	*P* value
Age (years)	86.5 ± 8.7	84.3 ± 11.6	0.016
Sex (male), n (%)	90 (55.9%)	1895 (55.2%)	0.861
Barthel score	44.5 ± 39.9	47.7 ± 40.4	0.332
CHADsVASC score	0.1 ± 0.7	0.1 ± 0.6	0.654
HASBLED score	3.5 ± 0.8	3.3 ± 1.1	0.074
HBA1c	4.0 ± 3.2	4.3 ± 4.0	0.281
**Complex chronic patients**			
Dementia cognitive impairment, n (%)	38 (23.6%)	720 (21.0%)	0.424
Diabetes, n (%)	58 (36.0%)	1452 (42.3%)	0.115
Atrial fibrillation, n (%)	51 (31.7%)	947 (27.6%)	0.257
Hipercholesterolemia, n (%)	89 (55.3%)	1628 (47.4%)	0.051
Ischemic cardiomyopathy, n (%)	33 (20.5%)	601 (17.5%)	0.331
Peripheral vascular disease, n (%)	17 (10.6%)	289 (8.4%)	0.342
Heart failure, n (%)	34 (21.1%)	913 (26.6%)	0.123
Thromboembolism, n (%)	13 (8.1%)	281 (8.2%)	0.960
Stroke or AIT, n (%)	16 (9.9%)	259 (7.5%)	0.264
Chronic renal disease, n (%)	36 (22.4%)	857 (25.0%)	0.455
Chronic liver disease, n (%)	1 (0.6%)	57 (1.7%)	0.306
Neoplasia, n (%)	18 (11.2%)	851 (24.8%)	< 0.001
**Medication use**			
Oral anticoagulant treatment, n (%)	54 (33.5%)	996 (29.0%)	0.217
Nonsteroidal antiinflammatory drugs, n (%)	112 (69.6%)	2611 (76.1%)	0.060
Antiaggregants, n (%)	103 (64.0%)	1817 (52.9%)	0.006
Statines, n (%)	85 (52.8%)	1921 (56.0%)	0.622

*ICH* Intracerebral hemorrhage, *HbA1c* Glycated hemoglobin, *CHADsVASC score* Congestive heart failure, hypertension, age ≥ 75y (doubled), diabetes mellitus, stroke (doubled)-vascular disease, age 65–74 and sex category (female), *HAS-BLEDscore* Hypertension, abnormal renal/liver function, stroke, bleeding history or predisposition, labile INR, elderly, drugs/alcohol concomitantly, *AIT* Transient ischemic attack

### Adjusted β (95% CI) for the association Association between HBA1c and Barthel score in patients with and without ICH

The univariate and multivariate linear regression analysis for association between HBA1c and Barthel score were performed in Table 2. Elevated HbA1c was positively and significantly related to a higher Barthel score among all study population (β = 1.25, 95% CI: 0.92, 1.59; *P* < 0.0001) with adjusted age and sex. Among non-ICH patients, increased HbA1c was still positively associated with an increased Barthel score (β = 1.24, 95% CI: 0.90, 1.58; *P* < 0.001). However, HbA1c appeared to have no any relationship with Barthel score in ICH patients (β = 1.87, 95% CI: -0.07, 3.82; *P* = 0.0613) after adjustment for age and sex. To avoid more confounding factors, we used sensitivity analysis to further confirm the independent association by adding CHADsVASC score, HAS-BLED score into the above linear model (Table 3). Interestingly, this strong relationship was still existed in non-ICH patients (β = 0.90, 95% CI: 0.56, 1.24; *P* < 0.001) but not in ICH patients (β = 1.88, 95% CI: -0.10, 3.86; *P* = 0.0649).

**Table 2:** The linear regression model for association between HbA1c and Barthel score in complex chronic patients

HBA1C	Model 1		Model 2	
	**β (95% CI)**	***P* value**	**β (95% CI)**	***P* value**
Total patients (*N* = 3594)	1.27 (0.93, 1.60)	< 0.0001	1.25 (0.92, 1.59)	< 0.0001
Patients with ICH (*N* = 161)	1.80 (-0.13, 3.73)	0.0689	1.87 (-0.07, 3.82)	0.0613
Patients without ICH (*N* = 3433)	1.26 (0.92, 1.60)	< 0.0001	1.24 (0.90, 1.58)	< 0.0001

Model 1: adjust for age

Model 2: Adjusted for age and sex

*ICH* Intracerebral hemorrhage

**Table 3:** Sensitivity analysis of linear regression model

HbA1c	Model 1		Model 2	
	**β (95% CI)**	***P* value**	**β (95% CI)**	***P* value**
Total patients (*N* = 3594)	0.92 (0.59, 1.26)	< 0.0001	0.92 (0.58, 1.26)	< 0.0001
Patients with ICH (*N* = 161)	1.79 (-0.17, 3.75)	0.0760	1.88 (-0.10, 3.86)	0.0649
Patients without ICH (*N* = 3433)	0.90 (0.56, 1.24)	< 0.0001	0.90 (0.56, 1.24)	< 0.0001

Model 1: adjust for age, CHADsVASC score and HASBLED score

Model 2: Adjusted for age, sex, CHADsVASC score and HASBLED score

*ICH* Intracerebral hemorrhage, *CHADsVASC score* Congestive heart failure, hypertension, age ≥ 75y (doubled), diabetes mellitus, stroke (doubled)-vascular disease, age 65–74 and sex category (female), *HAS-BLEDscore* Hypertension, abnormal renal/liver function, stroke, bleeding history or predisposition, labile INR, elderly, drugs/alcohol concomitantly

### Adjusted β (95% CI) for the association between HbA1c and Barthel score by subgroup analysis

In patients without stroke or TIA (β = 0.8, 95% CI: 0.5, 1.2; *P* < 0.001) in Table 4, we observed HbA1c was positively associated with Barthel score but not in those with stroke or TIA (β = 0.9, 95% CI: -0.6, 2.3; *P* = 0.234). Similar grouping differences were also existed in heart failure, atrial fibrillation, peripheral vascular disease, thromboembolism, chronic renal disease, neoplasia and medication use (Nonsteroidal antiinflammatory drugs). However, there were significant associations observed in patients with diabetes (β = 0.8, 95% CI: -0.4, 2.0; *P* = 0.197) and without diabetes (β = 0.3, 95% CI: -0.1, 0.7; *P* = 0.151).

**Table 4:** The stratified analysis for association between HbA1c and Barthel score in complex chronic patients

Covariates	Barthel score	
	**β (95% CI)**	***P* value**
**Dementia cognitive impairment**		
Yes	1.0 (0.2, 1.8)	0.015
No	0.8 (0.4, 1.2)	< 0.001
**Diabetes**		
Yes	0.8 (-0.4, 2.0)	0.197
No	0.3 (-0.1, 0.7)	0.151
**Atrial fibrillation**		
Yes	0.4 (-0.4, 1.2)	0.301
No	1.0 (0.6, 1.3)	< 0.001
**Hipercholesterolemia**		
Yes	0.7 (0.2, 1.2)	0.008
No	0.9 (0.4, 1.3)	< 0.001
**Ischemic cardiomyopathy**		
Yes	1.8 (0.8, 2.9)	< 0.001
No	0.7 (0.3, 1.0)	< 0.001
**Peripheral vascular disease**		
Yes	1.3 (-0.3, 2.9)	0.113
No	0.8 (0.4, 1.1)	< 0.001
**Heart failure**		
Yes	0.3 (-0.5, 1.1)	0.467
No	0.9 (0.5, 1.3)	< 0.001
**Thromboembolism**		
Yes	1.4 (-0.0, 2.8)	0.055
No	0.8 (0.5, 1.2)	< 0.001
**Stroke or TIA**		
Yes	0.9 (-0.6, 2.3)	0.234
No	0.8 (0.5, 1.2)	< 0.001
**Chronic renal disease**		
Yes	0.4 (-0.2, 1.0)	0.194
No	1.0 (0.6, 1.4)	< 0.001
**Chronic liver disease**		
Yes	NA	NA
No	0.9 (0.5, 1.2)	< 0.001
**Oral anticoagulant treatment**		
Yes	1.0 (0.3, 1.8)	0.008
No	0.9 (0.5, 1.2)	< 0.001
**Nonsteroidal antiinflammatory drugs**		
Yes	0.9 (0.5, 1.3)	< 0.001
No	0.5 (-0.3, 1.3)	0.241
**Antiaggregants**		
Yes	1.2 (0.6, 1.8)	< 0.001
No	0.6 (0.2, 1.0)	0.007
**Statines**		
Yes	0.7 (0.2, 1.2)	0.005
No	0.6 (0.1, 1.1)	0.012
**Neoplasia**		
Yes	1.2 (0.8, 1.7)	< 0.001
No	0.0 (-0.6, 0.6	0.903

Adjusted for age, sex, CHADsVASC score and HASBLED score

*AIT* Transient ischemic attack, *CHADsVASC score* Congestive heart failure, hypertension, age ≥ 75y (doubled), diabetes mellitus, stroke (doubled)-vascular disease, age 65–74 and sex category (female), *HAS-BLEDscore* Hypertension, abnormal renal/liver function, stroke, bleeding history or predisposition, labile INR, elderly, drugs/alcohol concomitantly

## Discussion

To our knowledge, this is first multi-center study with large sample size exploring the association between HbA1c and Barthel score that measure the functional state of patients' activities of daily living. We observed that elevated HbA1c levels were associated with better the activities of daily living evaluated by Barthel score, also after adjusted for confounding factors. Compared with patients with non-ICH, ICH did not increase the risk of poor prognosis, as well as stroke or TIA.

Existing research data have suggested that ICH is often has a high risk of hyperglycemia, and admission hyperglycemia has significant relation with adverse ICH outcomes and death risk [14–16]. From a pathophysiological perspective, hyperglycemia can be involved in increased perihematomal cell death and decreased autophagy to result in nerve injury, and increasing superoxide production and plasma kallikrein to promote hematoma enlargement [17–19]. These processes of which can be the causes of making or becoming the poor outcomes of ICH more serious. From a clinical perspective, numerous existing clinical studies have demonstrated that admission hyperglycemia is closely related to poor outcomes after ICH [4, 14–16]. For example, a previous INTERACT2 study consistently showed that diabetes or hyperglycemia were effective predictive factors for poor outcomes in individuals with mild or moderate-severe ICH [16]. A recent CHEERY study involving in a total of 1372 patients reported that stress-induced hyperglycemia was associated with a higher risk of pulmonary infection [risk ratios (RR): 1.477, 95% CI: (1.004, 2.172)], 30-day (RR: 1.068, 95% CI: 1.009–1.130) and 90-day mortality after ICH (RR: 1.060, 95% CI: 1.000–1.124) [4]. Inconsistent with our results, we found that HbA1c, a more stable indicator reflecting blood glucose levels, has a better daily life functions evaluated by Barthel score and diabetes did not also affect the results. The best explanation might be that the HbA1c levels of these ICH patients (4.0 ± 3.2) are almost all at normal levels, and reasonable blood sugar levels are also important indicators for maintaining the body's state in individuals with or without used hypoglycemic drugs. Besides, these study individuals were all non inpatient populations during a follow-up state. HbA1c levels not measured upon admission and relatively healthy physical condition may also be important factors leading to opposite outcomes.

However, opposite results were also existed in some previous study [10, 20–24]. A previous including 379 nonsurgical patients with spontaneous ICH with a follow-up for 3-month mortality, reported that a high admission plasma glucose level were not independent predictors of death risk [20]. In a study including a total of 228 consecutive adult patients with a mean age of 62.4 ± 12.9 years, there were no significant association observed between the random glucose levels (*r* = 0.108, *p* = 0.146) or fasting glucose levels (*r* = 0.116, *p* = 0.098) and functional outcomes at 90 days after discharge [21]. Moreover, a study cohort consisted of 237 patients who were presented to the emergency department with ICH and had blood glucose measured on presentation of emergency department, reporting that higher blood glucose on admission was associated with early mortality in both non-diabetics and diabetics (*P* < 0.0001), but the higher blood glucose was associated with poor functional outcome in non-diabetes patients (*P* < 0.0001) but not in diabetes patients (*P* = 0.268), which is completely consistent with our research results [22]. A prospective study included a total of 1,387 patients who had ICH and underwent brain computed tomography within 48 h of symptom onset at 33 centres in Korea between October 2002 and March 2004, suggested similar results that among patients without diabetes, glucose level had marginal significance for early (*p* = 0.053) and long-term mortality (*p* = 0.09) [23]. Another study included 2951 ICH patients with onset time ≤ 24 h from the China National Stroke Registry with a representative large cohort, also reporting similar association that elevated admission blood glucose confers a higher risk of 3-month poor outcome in non-diabetics than diabetics and the prognostic value of which was greater in non-diabetics than diabetics [24]. Hyperglycemia in most of these studies was measured based on admission blood glucose level, which may be caused by stress response from a transient hyperglycemic condition. This might explain why significant association between hyperglycemia and adverse outcomes tended to appear at nondiabetic patients rather than nondiabetic patients. In our study, among patients without ICH and stroke or TIA, we also observed HbA1c was positively associated with Barthel score, rather than in patients with ICH and stroke or TIA. These medical histories or stable ICH did not affected the independent association. Interestingly, a recent cohort study suggested a paradoxical relationship between HbA1c and in-hospital mortality in ICH patients. Their results showed that among 75,455 patients with ICH patients, patients with lower HbA1c (< 5.7%) had higher rates of in-hospital mortality in the entire cohort, as well as those with history of diabetes, and lower HbA1c was also associated with less chance of going home, and lower likelihood of having independent ambulatory status in patients with prior history of diabetes, which partly consistent with our results that a high HbA1c within a normal range is associated with improved prognosis [10]. The differences in this section mainly were stemed from numerous unknown and known confounding factors, including different study populations, sample sizes, analysis methods and other potential confounding.

A notable strength for our study was that we obtained data from a multi-center and population-based study with a large sample size (*N* = 3954) that ICH event has occurred in 161 individuals. As a supplement to the conflicting results of the relationship between HbA1c and adverse outcome in previous studies, we discovered for the first time that elevated HbA1c within a normal range was positively associated with improved ALD. This showed that in non diabetes, properly increasing the glycosylation level is related to the improvement of the patients' daily life function, which is consistent with existing basic studies. This significant association was still independent of potential confounding factors including age, sex, CHADsVASC score and HASBLED score by using linear regression analysis.

Yet, our study also has several limitations. Firstly, although the linear regression model could adjust some potential confounders, some other known and unknown confounding may still exist because unmeasured variables were not included into our analysis. Secondly, the retrospective analysis was observational in nature and still unable to confirm a causal relationship. Evidence should be based on more randomized clinical trial. Thirdly, our HbA1c value was not measured at admission, although it can reflect blood sugar levels in the past three or four months, which might lead to potential confounding in its relationship with patients' activities of daily living evaluated by Barthel score. Fourthly, we cannot clarify the classification of ICH because we had no information about pathological classification and location of ICH. There is a close relationship between different ICH severity and ADL, which is the focus of our next work.

## Conclusion

We came to the conclusion that elevated HbA1c is associated with better ADL in CCPs without ICH but not in those with ICH, which might provide new insight for HbA1c and its associations with prognosis of patients with chronic diseases.

## Data Availability

We obtained the dataset that has been deposited in the public repository (10.5061/dryad.t76hdr7zj).
